# Procyanidin B1 and Coumaric Acid from Highland Barley Alleviated High-Fat-Diet-Induced Hyperlipidemia by Regulating PPARα-Mediated Hepatic Lipid Metabolism and Gut Microbiota in Diabetic C57BL/6J Mice

**DOI:** 10.3390/foods13121843

**Published:** 2024-06-12

**Authors:** Zehua Liu, Jianshen Liu, Ruoxin Tang, Zhaowan Zhang, Shuangqi Tian

**Affiliations:** 1 Grain, Oil and Food Engineering Technology Research Center of the State Grain and Reserves Administration/Key Laboratory of Henan Province, College of Food Science and Technology, Henan University of Technology, Zhengzhou 450001, China; jctiansd@126.com (J.L.); 18638707683@163.com (R.T.); zhangzhaowan1616@163.com (Z.Z.); tianshuangqi@haut.edu.cn (S.T.); 2 Food Laboratory of Zhongyuan, Henan University of Technology, Zhengzhou 450001, China

**Keywords:** whole-grain highland barley, procyanidin B1, coumaric acid, high-fat diet, hyperlipidemia, diabetes, gut microbiota dysbiosis

## Abstract

A whole-grain highland barley (WHB) diet has been recognized to exhibit the potential for alleviating hyperlipidemia, which is mainly characterized by lipids accumulation in the serum and liver. Previously, procyanidin B1 (PB) and coumaric acid (CA) from WHB were found to alleviate serum lipid accumulation in impaired glucose tolerance mice, while the effect on modulating the hepatic lipid metabolism remains unknown. In this study, the results showed the supplementation of PB and CA activated the expression of peroxisome proliferator-activated receptor α (PPARα) and the target genes of cholesterol 7-α hydroxylase (*CYP7A1*) and carnitine palmitoyl transferase I (*Cpt1*) in the liver cells of high-fat-diet (HFD)-induced diabetic C57BL/6J mice, resulting in decreases in the serum total cholesterol (TC), triglyceride (TG), and low-density lipoprotein (LDL-C) contents, and an increase in the high-density lipoprotein (HDL-C) content. High-throughput sequencing of 16S rRNA indicated that supplementation with PB and CA ameliorated the gut microbiota dysbiosis, which was associated with a reduction in the relative abundance of *Ruminococcaceae* and an increase in the relative abundance of *Lactobacillus, Desulfovibrio*, and *Akkermansia*. Spearman’s correlation analysis revealed that these genera were closely related to obesity-related indices. In summary, the activation of PPARα expression by PB and CA from WHB was important for the alleviation of hyperlipidemia and the structural adjustment of the gut microbiota.

## 1. Introduction

The long-term consumption of a high-fat diet (HFD) is an important contributor to hyperlipidemia, which is a serious risk for the development of metabolic syndromes such as obesity and type 2 diabetes [1,2]. The liver serves as a pivotal organ, playing a crucial role in the synthesis, metabolism, and transport of lipids and peroxisome proliferator-activated receptor alpha (PPARα) is an important target of lipid metabolism and primarily expressed in the liver, in which it participates in β-oxidation and fatty acid transport, while cholesterol 7-α hydroxylase (*CYP7A1*) and carnitine palmitoyl transferase I (*Cpt1*) were found to be the target genes of PPARα [3,4,5]. With increasing attention on HFD-induced hyperlipidemia, dietary intervention with functional foods or functional ingredients has been emphasized and considered to be initiated for the alleviation of hyperlipidemia. It was reported that dietary polyphenols such as catechin, (-)-epicatechin gallate and (-)-epigallocatechin-3-gallate from green tea could regulate Cyp7a1 protein levels and cause reductions in liver cholesterol (TC), low-density lipoprotein (LDL), and triglyceride (TG) contents, thereby alleviating hyperlipidemia [6,7]. Dietary phenolic compounds from pickled *Raphanus sativus* L. could inhibit lipid accumulation in the liver of obese mice and decrease the TC and TG contents [8]. In addition, to positively regulate the gene expression of the hepatic lipid metabolism, activated PPARα can negatively interfere with the activity of proinflammatory transcription factors resulting in the participation of an anti-inflammatory effect [1]. Therefore, the use of dietary polyphenols is considered an effective strategy in alleviating hyperlipidemia.

Highland barley (*Hordeum vulgare* L.; HB) is a hull-less barley cultivar mainly distributed in highland areas, which is rich in components, including 3.7–7.7% -glucans, 11.5–14.2% proteins, 4.7–6.8% lipids, and phenolic compounds such as catechin, chlorogenic acid, and p-coumaric acid [9,10]. Previous studies have demonstrated that HB grains and their phenolic compound extracts effectively mitigate the severity of hyperlipidemia, regulate lipid metabolism, enhance antioxidant stress, and alleviate liver inflammation [11,12,13,14]. However, the active compounds that contribute to the modulation of hyperlipidemia are still little known. In a previous study by our research team, we found that procyanidin B1 (PB) and coumaric acid (CA) extracted and separated from whole-grain HB exhibited noticeable effects on decreasing the serum TC, TG, and LDL-C contents and increasing the high-density lipoprotein cholesterol (HDL-C) content in impaired glucose tolerance mice [10]. Notably, the combination of PB and CA indicated a potential synergistic effect. Therefore, in this study, we aimed at exploring whether PB and CA could alleviate hyperlipidemia and regulate the hepatic lipid metabolism via activating PPARα expression.

On the other hand, it has been recognized that functional foods, along with their bioactive constituents, can modify the composition of the gut microbiota, which may contribute to the alleviation of hyperlipidemia [15,16]. Previous studies reported that the consumption of a whole-grain diet showed potential for preventing hyperlipidemia in people [17,18]. Animal studies have demonstrated that the consumption of an HB diet reduces the risk of hyperlipidemia and is closely associated with changes in the gut microbiota [11,12,19,20,21,22]. The β-glucan from HB was reported as an active component that could help improve gut microbiota dysbiosis [23,24]. However, the role HB phenolic compounds play in the modulation of gut microbiota dysbiosis is still rarely understood. Additionally, it has been reported that genera such as *Lactobacillus*, *Allobaculum*, *Akkermansia*, and *Bifidobacterium* could improve liver inflammation, and *Bifidobacterium* and *Lactobacillus* in the gut play a significant role in the activities of β-glucosidase and bile salt hydrolase enzymes, which are capable of converting anthocyanins into phenolic acids, producing short-chain fatty acids (SCFAs), reducing serum cholesterol levels, and increasing the excretion of sterols in the feces [19,25,26]. Previous reports also highlighted PPARα’s key role between microbial metabolism and epithelial repair in the virus-inflamed gut, and as a potential mitochondrial target for restoring the gut barrier [27]. Morais et al. found that the supplementation of a trans-fatty acid-based diet with rich-in-anthocyanins chrysanthemum powder in rats tended to normalize weight gain, increase the serum TC and TG contents, and restore the populations of *Bifidobacterium* and *Lactobacillus* in the gut [26]. Based on this, we supposed that PB and CA from whole-grain HB may improve gut microbiota dysbiosis and be helpful for the alleviation of hyperlipidemia. Therefore, in this study, we investigated the effects of PB and CA from whole-grain HB on the alleviation of high-fat-diet-induced hyperlipidemia by regulating the PPARα-mediated hepatic lipid metabolism and gut microbiota in diabetic C57BL/6J mice.

## 2. Materials and Methods

### 2.1. Reagents and Raw Materials

Total cholesterol (Kit No. A111-1-1), triglyceride (Kit No. A110-1-1), high-density lipoprotein cholesterol (HDL-C) (Kit No. A112-1-1), low-density lipoprotein cholesterol (LDL-C) (Kit No. A113-1-1), aspartate aminotransferase (AST) (Kit No. C010-2-1), alanine aminotransferase (ALT) (Kit No. C009-2-1), malondialdehyde (MDA) (Kit No. A003-4-1), tumor necrosis factor-α (TNF-α) (Kit No. H052-1-2), interleukin-1β (IL-1β) (Kit No. H002-1-2), and interleukin-6 (IL-6) (Kit No. H007-1-2) assay kits were purchased from Nanjing Jiancheng Biomedical Company (Nanjing, Jiangsu, China). Chemical regents were obtained from Solarbio Co., Ltd. (Beijing, China). All procedures were conducted according to the manufacturers’ instructions. Antibodies of β-actin, PPARα, Cpt1b, and Cyp7a1 were purchased from Proteintech Group, Inc. (Wuhan, China).

The compounds procyanidin B1 and p-coumaric acid from highland barley (cultivated from Qinghai Province, variety of Beiqing 3) were extracted and separated according to the method used in our laboratory and reported in our previous study, with procyanidin B1 and p-coumaric acid both being 92% pure [10].

### 2.2. Animal Care

Male C57BL/6J mice (4 weeks of age, weighing 15 ± 0.31 g, total of 95 mice) were purchased from the Animal Experimental Center of Zhengzhou University (Zhengzhou, China) and maintained in an air-conditioned room (21–23 °C and 42–66% humidity) with a 12 h light/dark cycle (4 mice per cage). After 1 week of acclimation, mice were fed either a low-fat diet (normal group (LFD); rat and mouse maintenance diet 1025; Beijing HFK Bioscience Co., Ltd., Beijing, China) or a high-fat diet (model group (HFD); D12492, 60% kcal% fat; Research Diets, New Brunswick, NJ, USA). Following HFD feeding for 4 weeks, the model group (M) mice fed with HFD were injected with streptozocin (STZ) (30 mg/kg body weight, intraperitoneally, in citrate buffer (pH 4.5)), and the normal group mice (N) were injected with the same volume of saline. The mice that maintained fasting blood glucose above 7.0 mM and postprandial blood glucose above 11.1 mM were considered to display impaired glucose tolerance and were selected for further studies [10]. The total number of mice used for further studies including LFD feeding and HFD feeding was 88.

### 2.3. Dosage Information

**Figure 1:**
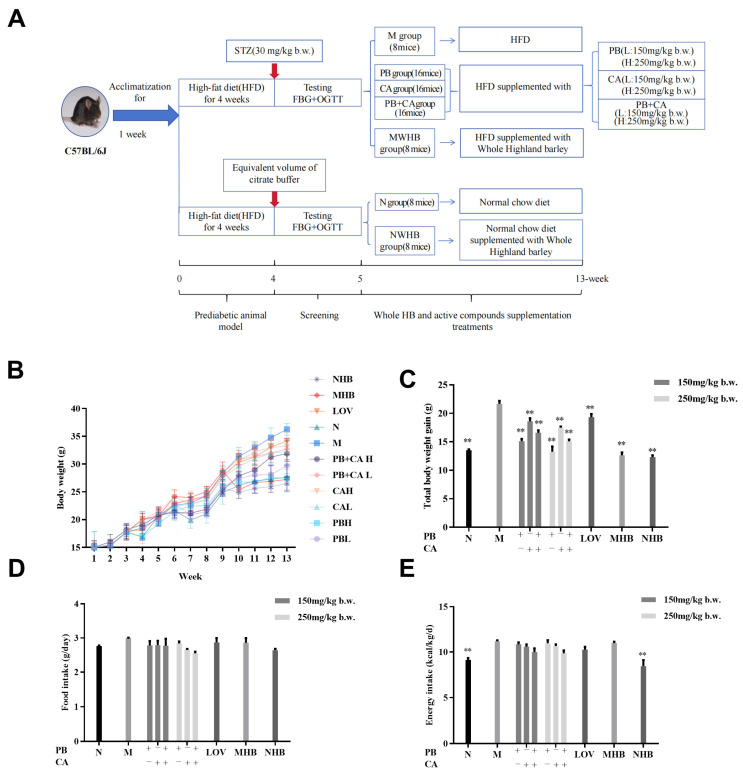
Procyanidin B1 and coumaric acid from whole-grain highland barley reduced body weight in HFD-fed mice. (**A**) Experimental protocol and design; (**B**) changes in body weight over 12 weeks and (**C**) body weight gain; (**D**) mean food intake and (**E**) mean energy intake. Data are shown as means ± SEM, with n = 8 mice per group. Significant differences between groups and model group (M) were analyzed with one-way ANOVA with Dunnett’s multiple comparison test and are indicated by ** for *p* < 0.01. PBL and PBH: model group supplemented with procyanidin B1 at low and high dosages, respectively; CAL and CAH: model group supplemented with coumaric acid at low and high dosages, respectively; PB + CA L and PB + CA H: model group supplemented with combination of procyanidin B1 and coumaric acid at low and high dosages, respectively; M: model group; N: normal group; MHB and NHB: model group and normal group supplemented with whole-grain highland barley, respectively; LOV: model group supplemented with lovastatin.

The mice of the bioactive compound-treated groups (8 mice per group) were supplemented daily for 8 weeks with procyanidin B1 (PB), p-coumaric acid (CA), and procyanidin B1 + p-coumaric acid (PB + CA; 1:1 *w*/*w*) at a low dosage of 150 mg/kg b.w. (named PBL, CAL, and PB + CAL, respectively) and a high dosage of 250 mg/kg b.w. (PBH, CAH, and PB + CAH, respectively) [10,28]. The human-equivalent doses for the bioactive compound-treated groups were 16.65 mg/kg b.w. for the low dosage and 27.75 mg/kg b.w. for the high dosage, respectively. The mice of the positive control group were model mice given 0.03% lovastatin (named LOV) [18,20]. The MHB group and NHB group were model mice and normal mice, respectively, fed with 25 g of whole-grain highland barley (uniform mixed into the corresponding chow diet and to keep the final chow diet weight intake of mice the same for the model and normal groups, respectively, there was an equivalent dose of approximately 200 mg/kg b.w. active compounds). The mice in the M, N, MHB, and NHB groups were given the same volume of saline (Figure 1A). Food intake and body weight were measured weekly. At the end of this experiment, all mice were fasted for 12 h to obtain the blood samples from the orbital vascular plexus before sacrifice. The serum was separated by centrifugation at 3000 rpm for 10 min and stored at −80 °C until analysis. The liver and adipose tissues were harvested, weighed, snap-frozen in liquid nitrogen, and then stored at −80 °C. All experimental procedures were performed according to the standard guidelines for animal care and approved by the Welfare Committee of Animal Experimental Centre, Zhengzhou, China (approval No. zzu-LAC20230306[01]).

### 2.4. Determination of Serum Lipids and Hepatic AST and ALT Activities

Serum TC, TG, LDL-C, and HDL-C contents were measured by using the corresponding kits and following the instructions. Liver tissues (100 mg) were homogenized in 1 mL of PBS, and AST and ALT activities were determined by using the corresponding kits and following the instructions; their final values were expressed as U/L.

### 2.5. Histopathological Study

Liver and adipose samples fixed in 10% neutral formalin were dehydrated through successive passing by using a gradient of mixtures of ethyl alcohol and water. They were embedded in paraffin after being rinsed with xylene. The liver and adipose sections (5 μm thick) were stained with hematoxylin and eosin for evaluation by using light microscopy (microscope magnification was 200×). The cell size of the adipocytes was quantified by Image J software (Rasband, W.S., ImageJ, U. S. National Institutes of Health, Bethesda, MD, USA), and the cell size was calculated as the average value of 5 areas randomly selected in the slides [29].

### 2.6. Immunohistochemistry Analysis

The liver tissues of the treated mice were fixed in PBS containing 4% paraformaldehyde for 1 h; then, they were embedded in OCT compound (Sakura Finetek, Tokyo, Japan) and stored at −80 °C. Afterwards, the tissues were incubated with 3% H_2_O_2_ for 10 min and blocked in 5% bovine serum albumin (from Sigma-Aldrich (Shanghai) Trading Co., Ltd.) for 1 h. Then, primary antibody was added to the tissues and incubated overnight. The slides were washed with PBS three times and probed with secondary antibody for 1 h. The slides were washed again with PBS 3 times and stained with DAB brown staining solution (Nacalai Tesque) for 10 min. The sections (5 μm thick) were counterstained with hematoxylin and dehydrated in a series of alcohols. The immunoperoxidase changes were captured by using an inverted light microscope (Leica), and protein expression was quantified by using ImageJ software.

### 2.7. Western Blot Analysis

Western blot analysis was conducted as described by Liu et al. with slight modifications [28]. In brief, the total protein of liver tissues was isolated with lysis buffer; then, the supernatants were collected, and the protein contents were detected by using BCA reagent. Subsequently, equal contents of protein were separated by using 10% sodium dodecyl sulfate polyacrylamide gel electrophoresis and transferred onto polyvinylidene difluoride membranes. After being blocked with 5% nonfat milk, the membranes were incubated with different primary antibodies, i.e., PPARα, Cpt1b, and Cyp7a1 (1:1000 dilution), at 4 °C overnight. Afterwards, the membranes were incubated for 1 h at room temperature with horseradish peroxidase-conjugated secondary antibodies (1:10,000 dilution). The immunocomplexes were finally observed with a UVP BioSpectrum^®^ 415 Imaging System (Upland, CA, USA), and β-actin was used as an internal control.

### 2.8. Quantitative RT-PCR Assay

Total RNA isolation, cDNA reverse transcription, and RT-PCR assays were conducted according to the procedure described by Deng et al. with slight modifications [19]. Briefly, total RNA was extracted from the liver tissue with TRIzol reagent. Afterwards, total RNA was synthesized to cDNA with a PrimeScript RT Reagent kit (Takara Biotechnology, Dalian, China). Total reaction volume was 20 μL, including 1 μg of total RNA, 4 μL of 5 × transcript all-in-one supermix for qPCR, 1 μL of gDNA remover, and 14 μL of RNase-free water. RT-PCR was conducted on a Bio-Rad MiniOpticon real-time PCR system (Bio-Rad, CA, USA) by using cDNA, forward and reverse primers, and SYBR Green Supermix (TIANGEN Biotech, Beijing, China). The total reaction volume was 20 μL, including 10 μL of qPCR Supermix solution, 2 μL of forward and reverse primers, 2 μL of DNA template, and 6 μL of distilled water. The sequences of the primers were adopted as Hou et al. reported previously [30]. Beta-actin was assigned to calibrate the relative expression of mRNA. The final results were calculated by using the 2^−ΔΔCT^ method.

### 2.9. Gut Microbiota Profiling

The total genome DNA of bacteria was extracted with a QIAamp DNA stool Mini Kit (Qiagen, Hilden, Germany) from frozen feces according to the manufacturer’s instructions. The 16S rDNA gene was amplified by using a specific primer with the barcode (16S V3 + V4). DNA sequencing libraries were constructed by using a TruSeq^®^ DNA PCR-Free Sample Preparation Kit (Suzhou RENOLD Biological Technology Co., Ltd., China). Standard thermal cycling (95 °C for 5 min (1 cycle), 95 °C for 30 s, 50 °C for 30 s, and 72 °C for 40 s (25 cycles)) and extension (72 °C for 7 min) conditions were used for PCR amplification in the presence of Fast Hifidelity Polymerase and Phusion^®^ High-Fidelity PCR Master Mix with GC Buffer (New England Biolabs Co., Ltd., Beijing, China). The paired-end sequencing of the PCR products was performed with a NovaSeq6000 at Suzhou Bionovogene Co., Ltd. (Suzhou, China) [31].

### 2.10. Statistical Analysis

Data are expressed as means ± standard deviations (n = 8). Unpaired Student’s *t* tests were used for comparisons between two groups. Comparisons between the control group (model group) and the supplemented groups were assessed by using one-way ANOVA with Dunnett’s *t*-test on SPSS (SPSS version 17.0; IBM Inc., Armonk, NY, USA). Differences were considered statistically significant when *p* < 0.05 and very significant when *p* < 0.01. Spearman’s nonparametric correlation between the gut microbiota and HFD-related parameters was performed using R software (version 3.5.1, Centers for Disease Control and Prevention, Atlanta, GA, USA).

## 3. Results

### 3.1. Procyanidin B1 and Coumaric acid Attenuated HFD-Induced Weight Gain

After 12 weeks of feeding, compared with the normal group (N), the body weight gain of the mice fed with the HFD (M) showed a significant increase (Figure 1B,C). In contrast, procyanidin B1 (PB) and coumaric acid (CA) supplementation significantly inhibited the HFD-induced body weight gain in a dose-dependent manner (*p* < 0.05). The average food intake (Figure 1D) and energy intake (Figure 1E) of the mice were measured to determine whether the supplementation with PB and CA affected mouse behavior through the regulation of food intake or appetite. It is noteworthy that there were no significant differences in food intake and energy intake between the M group and the PB groups, nor between the CA groups and the PB + CA groups, indicating that these factors were not the primary causes of the weight loss observed.

### 3.2. Procyanidin B1 and Coumaric Acid Ameliorated HFD-Induced Inflammation and Lipid Disorders in Serum

**Figure 2:**
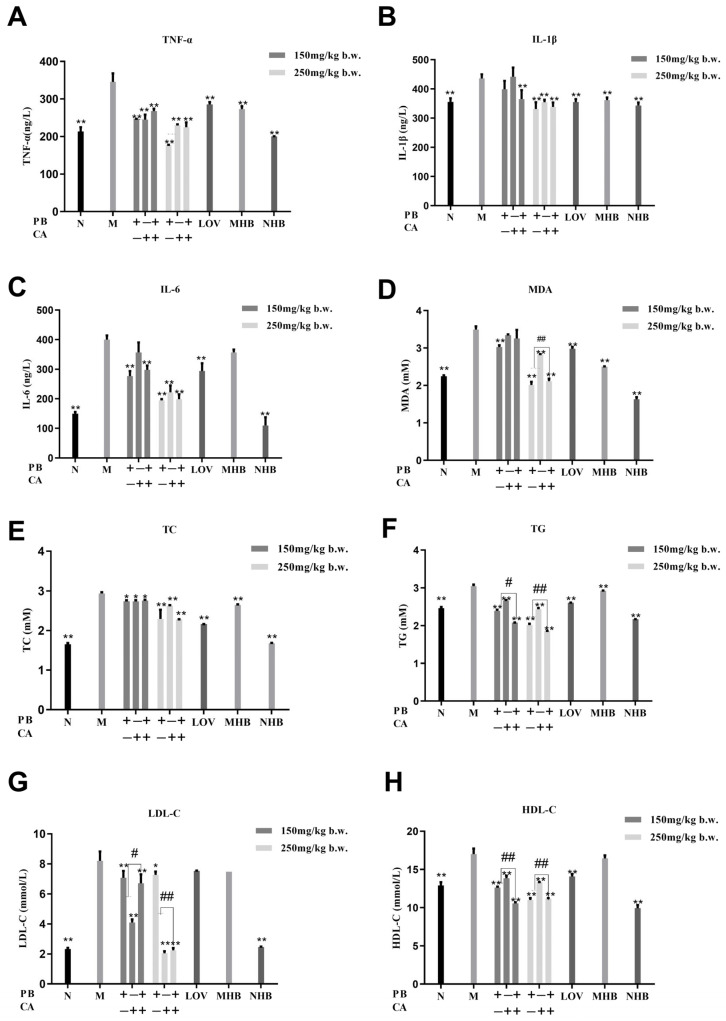
Procyanidin B1 and coumaric acid inhibited HFD-induced inflammatory response and lipid disorders in serum. Serum (**A**) TNF-α, (**B**) IL-1β, (**C**) IL-6, (**D**) MDA, (**E**) TC, (**F**) TG, (**G**) LDL-C, and (**H**) HDL-C contents. Data are shown as means ± SEM, with n = 8 mice per group. Significant differences between groups and model group (M) were analyzed with one-way ANOVA with Dunnett’s multiple comparison test and are indicated by * for *p* < 0.05 and ** for *p* < 0.01. Significant differences compared with value of combination groups (PB + CA) and average value of PB and CA groups were analyzed with unpaired Student’s *t* test and are indicated by ^#^ for *p* < 0.05 and ^##^ for *p* < 0.01. PBL and PBH: model group supplemented with procyanidin B1 at low and high dosages, respectively; CAL and CAH: model group supplemented with coumaric acid at low and high dosages, respectively; M: model group; N: normal group; MHB and NHB: model group and normal group supplemented with whole-grain highland barley, respectively; LOV: model group supplemented with lovastatin.

Supplementation with PB, CA, and PB + CA is hypothesized to effectively improve the serum inflammation and lipid disorders induced by a high-fat diet (HFD), given their significant benefits in weight control. The levels of inflammatory cytokines, including TNF-α, IL-1β, and IL-6, were measured, and the results reveal that PB, CA, and PB + CA significantly reduced the levels of these inflammatory cytokines in mouse serum (Figure 2A–C) (*p* < 0.01). Notably, the high-dosage supplementation with PB, CA, and PB + CA normalized the serum TNF-α, IL-1β, and IL-6 contents to similar levels to the N group, suggesting that PB, CA, and PB + CA supplementation could markedly ameliorate HFD-induced systemic low-grade inflammation.

Further, the serum lipid profiles were determined to explore the effects of PB, CA, and PB + CA on alleviating hyperlipidemia. As expected, feeding mice an HFD for 12 weeks resulted in a significant increase in the serum levels of MDA, TC, TG, and LDL-C compared with the N group (Figure 2D–G). However, the supplementation with PB, CA, and the combination of PB + CA was found to significantly reduce the lipid contents in the serum in a dose-dependent manner (*p* < 0.05). Notably, the combination of PB and CA showed synergistic effects on decreasing the serum MDA content at a high dosage and serum TG and LDL-C contents at both low and high dosages (*p* < 0.05), since the related values of the combination group were significantly lower than the average value of the individual groups. On the other hand, the supplementation with PB, CA, and PB + CA significantly increased the serum HDL-C contents (Figure 2H) (*p* < 0.01), and the combination of PB and CA also showed synergistic effects on improving the serum HDL-C content at a high dosage. These results indicate that supplementation with PB and CA has strong effects on alleviating serum lipid deposition, with the combination of the two compounds achieving synergistic impacts.

### 3.3. Procyanidin B1 and Coumaric Acid Reduced HFD-Induced Hepatic Steatosis and Adipose Tissue Hypertrophy

**Figure 3:**
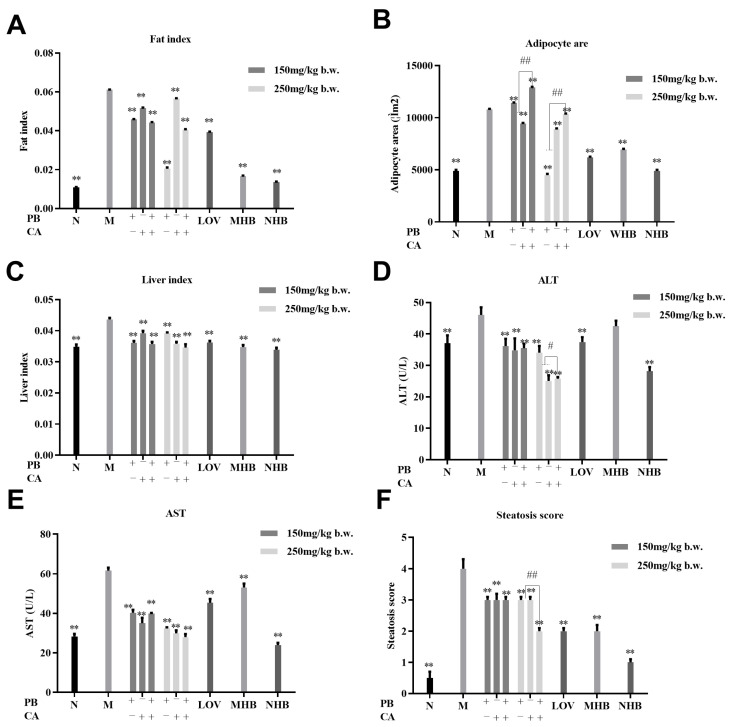
Procyanidin B1 and coumaric acid synergistic alleviated adipose and liver histopathological changes in HFD-fed mice. (**A**) Abdominal subcutaneous fat index; (**B**) adipocyte area; (**C**) liver index; (**D**,**E**) ALT and AST activities in liver; (**F**) steatosis score of liver; H&E staining of abdominal subcutaneous fat and liver tissues. Liver and fat indexes were calculated as [tissue mass (g)/body weight (g)]. Data are shown as means ± SEM with n = 8 mice per group. Significant differences between groups and model group (M) were analyzed with one-way ANOVA with Dunnett’s multiple comparison test and are indicated by ** for *p* < 0.01. Significant differences compared with value of combination groups (PB + CA) and average value of PB and CA groups were analyzed with unpaired Student’s *t* test and are indicated by ^#^ for *p* < 0.05 and ^##^ for *p* < 0.01. PBL and PBH: model group supplemented with procyanidin B1 at low and high dosages, respectively; CAL and CAH: model group supplemented with coumaric acid at low and high dosages, respectively; M: model group; N: normal group; MHB and NHB: model group and normal group supplemented with whole-grain highland barley, respectively; LOV: model group supplemented with lovastatin.

**Figure 4:**
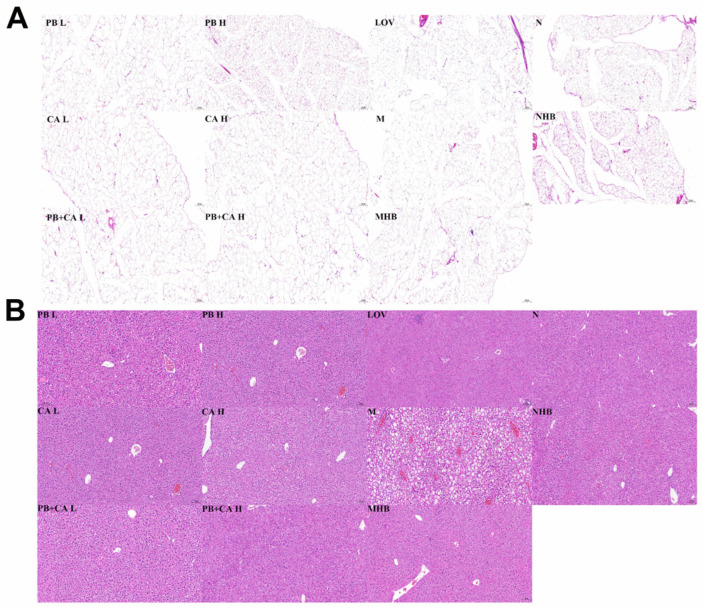
H&E staining of abdominal subcutaneous fat (**A**) and liver tissues (**B**) of procyanidin B1- and coumaric acid-supplemented HFD-fed mice. Liver and fat indexes were calculated as [tissue mass (g)/body weight (g)]. PBL and PBH: model group supplemented with procyanidin B1 at low and high dosages, respectively; CAL and CAH: model group supplemented with coumaric acid at low and high dosages, respectively; PB + CA L and PB + CA H: model group supplemented with combination of procyanidin B1 and coumaric acid at low and high dosages, respectively; M: model group; N: normal group; MHB and NHB: model group and normal group supplemented with whole-grain highland barley, respectively; LOV: model group supplemented with lovastatin.

As shown in Figure 3A, the abdominal subcutaneous fat index was significantly decreased after supplementation with PB and CA, which was consistent with the changes in body weight (*p* < 0.01). The M group showed significantly more abnormal lipid accumulation in adipose tissue; however, the PB, CA, and PB + CA supplementation significantly suppressed the severe hypertrophy of adipocytes (Figure 3B and Figure 4A) in a dose-dependent manner.

As shown in Figure 3C, the supplementation with PB and CA also led to a reduction in the liver index. Furthermore, severe hepatocyte ballooning degeneration, hepatic vacuoles, and inflammation infiltrates were observed in the M group compared with the N group in Figure 4B. These effects were significantly mitigated following the supplementation with PB, CA, and PB + CA, as evidenced by the levels of AST and ALT (Figure 3D,E) and the liver steatosis score (Figure 3F). Notably, the combination of PB and CA at a high dosage showed synergistic effects on the steatosis score, suggesting that the combination of PB and CA may achieve stronger effects on alleviating hepatic steatosis.

### 3.4. Procyanidin B1 and Coumaric Acid Activated PPARα Expression in Liver

**Figure 5:**
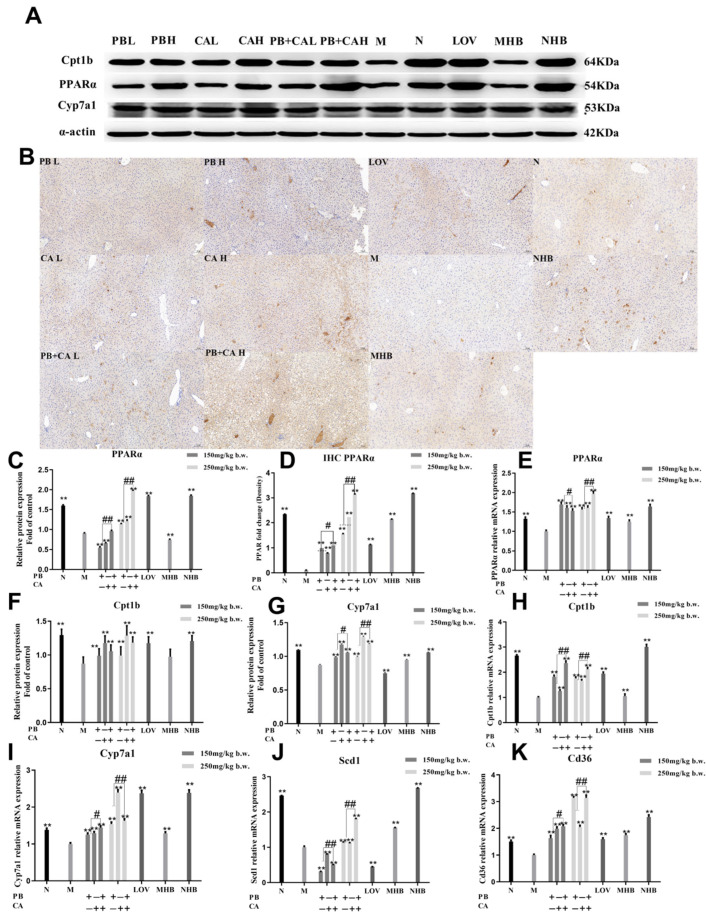
Effects of procyanidin B1 and coumaric acid on protein and gene expression of PPARα. (**A**) Representative image and corresponding fold changes in (**C**) PPARα, (**F**) Cpt1b, and (**G**) Cyp7a1 protein expression according to Western blot analysis. (**B**) Immunohistochemistry of PPARα protein expression in mouse liver cells and (**D**) fold change in relative densitometric levels of PPARα. (**E**) Gene expression of *PPARα* and target gene expression of (**H**) *Cpt1b*, (**I**) *Cyp7a1*, (**J**) *Scd1*, and (**K**) *Cd36* according to RT-PCR analysis. Data are shown as means ± SEM, with n = 8 mice per group. Significant differences between groups and model group (M) were analyzed with one-way ANOVA with Dunnett’s multiple comparison test and are indicated by ** for *p* < 0.01. Significant differences compared with value of combination groups (PB + CA) and average value of PB and CA groups were analyzed with unpaired Student’s *t* test and are indicated by ^#^ for *p* < 0.05 and ^##^ for *p* < 0.01. PBL and PBH: model group supplemented with procyanidin B1 at low and high dosages, respectively; CAL and CAH: model group supplemented with coumaric acid at low and high dosages, respectively; PB + CA L and PB + CA H: model group supplemented with combination of procyanidin B1 and coumaric acid at low and high dosages, respectively; M: model group; N: normal group; MHB and NHB: model group and normal group supplemented with whole-grain highland barley, respectively; LOV: model group supplemented with lovastatin.

To further explore the related pathways of PB, CA, and PB + CA in the modulation of HFD-induced hyperlipidemia in mice, the expression of the PPARα protein and that of its target genes were determined. As shown in Figure 5A–C, the PPARα expression level in the liver cells of the HDF-induced mice was abnormally low. However, after the supplementation with PB, CA, and PB + CA, the protein expression of PPARα was significantly increased (*p* < 0.01). Consistently, the immunohistochemistry and RT-PCR analyses confirmed that the supplementation with PB and CA significantly up-regulated the protein expression of PPARα, as well as the gene expression (Figure 5D,E). Notably, the combination of PB and CA significantly improved the PPARα protein and gene expression compared with PB and CA (*p* < 0.05) at both low and high dosages.

Moreover, supplementation with PB and CA significantly increased the protein and gene expression of Cpt1b (Figure 5F,H), with the combination of the two compounds exhibiting synergistic effects on the up-regulation of Cpt1b gene expression (*p* < 0.05). Similarly, HFD feeding significantly reduced the protein and gene expression of Cyp7a1, the target gene of PPARs, which plays a key role in cholesterol synthesis (Figure 5G,I). The supplementation with PB and CA significantly improved the protein and gene expression of Cyp7a1 and exhibited synergistic effects (*p* < 0.01). In contrast, the supplementation with PB, CA, and PB + CA significantly reduced the increase in HFD-induced *Scd1* and *Cd36* gene expression (Figure 5J,K). The combination of PB and CA showed significant differences in decreasing the Cd36 gene expression compared with individual PB and CA administration (*p* < 0.01). These results indicate that supplementation with PB and CA may alleviate hyperlipidemia by regulating *Cpt1b* and *Cyp7a1* gene expression, thus reducing PPARα-mediated hepatic steatosis, as well as *Scd1* and *Cd36* gene expression, thus reducing hepatic lipogenesis.

### 3.5. Procyanidin B1 and Coumaric Acid Restored HFD-Induced Gut Microbiota Dysbiosis

To assess the impact of supplementation with PB and CA on the intestinal microbiota dysbiosis induced by a high-fat diet (HFD), high-throughput sequencing based on the V3–V4 region of the 16S rRNA gene from fecal bacteria was conducted to analyze the changes in intestinal microbiota composition. After quality filtering, chimera checking, and removal of low-quality reads, the average cleaned reads for the 33 samples were at least 37,091.

**Figure 6:**
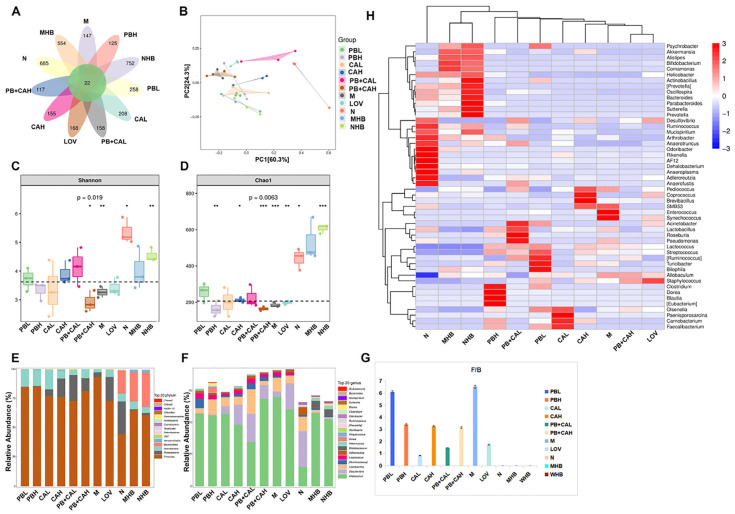
Protective effects of procyanidin B1 and coumaric acid from highland barley on gut microbiota. (**A**) Venn diagrams; (**B**) principal coordinates analysis (PcoA) based on OTU level; (**C**,**D**) Shannon and Chao1 index; (**E**,**F**) phylum- and genera-level fecal microbiota; (**G**) relative abundance of Firmicutes-to-Bacteroidetes ratio at phylum level; (**H**) heatmap analysis at genera level. Statistical differences between the M group and each treatment group were analyzed with the *t*-test. PBL and PBH: model group supplemented with procyanidin B1 at low and high dosages, respectively; CAL and CAH: model group supplemented with coumaric acid at low and high dosages, respectively; PB + CA L and PB + CA H: model group supplemented with combination of procyanidin B1 and coumaric acid at low and high dosages, respectively; M: model group; N: normal group; MHB and NHB: model group and normal group supplemented with whole-grain highland barley, respectively; LOV: model group supplemented with lovastatin. Significant differences were indicated by * for *p* < 0.05, ** for *p* < 0.01 and *** for *p*< 0.001.

The Venn diagrams reveal that compared with the diet of the N group, the HFD reduced the number of OTUs in the intestinal microbiota and that supplementation with PB and CA reversed this decline (Figure 6A). Consistently with these findings, the supplementation with PB and CA significantly increased the Shannon and Chao1 indexes (Figure 6C,D), indicating an enhancement in the diversity and richness of the microbiota community. Alpha diversity analysis demonstrated that the supplementation with PB and CA may help protect the decrease in microbial richness caused by the HFD. However, no statistically significant differences were observed in the Shannon and Chao1 indexes among these groups. The beta diversity values, calculated by using principal coordinate analysis based on OTU abundance, further indicated that the composition of the intestinal microbiota in the HFD group was completely different from that in the N group. The intestinal microbiota composition in response to the supplementation with PB, CA, and PB + CA was significantly responsive to the HFD (Figure 6B). Although the aggregation of the PC and PB groups at a high dosage was close to that of the M group, a more distinct clustering and a more remarkable change in the gut microbial structure was observed compared with the M group. Notably, the MHB and NHB groups exhibited significant distinct clustering compared with the other groups, indicating that whole-grain highland barley may induce specific changes in the microbial structure. The combination of PB and CA at a low dosage (150 mg/kg b.w.) induced changes in the gut microbial structure close to those in the N group, suggesting that PB + CA may exert effects on the microbial structure similar to those observed in the N group.

To determine significant changes in the composition of the intestinal microbiota associated with the supplementation with PB, CA, and their combination (PB + CA), the relative abundance of the top 20 groups at the phylum and genera level were determined. As shown in Figure 6E, the mouse intestinal microbiota was predominantly composed of Firmicutes and Bacteroidetes. Compared with the N group, the relative abundance of Firmicutes was significantly increased in the M group; however, this was reversed with the supplementation with PB and CA. Compared with Firmicutes, the relative abundance of Bacteroidetes showed a stark contrast, with the supplementation with PB, CA, and PB + CA decreasing the Firmicutes/Bacteroidetes (F/B) ratio induced by the high-fat diet (HFD) (Figure 6G). At the phylum level, HFD feeding significantly increased the relative abundance of *Firmicutes* and decreased that of *Actinobacteria*, *Proteobacteria*, *Verrucomicrobia*, and *Bacteroidota*, while the supplementation with PB and CA reversed such effects (Figure 6E). At the genera level, HFD feeding significantly increased the relative abundance of *Allobaculum* and decreased that of *Lactobacillus*, *Ruminococcus*, *Adlercreutzia*, and *Desulfovibrionales*, while the effect was reversed with the supplementation with PB and CA (Figure 6F). Notably, the combination of PB and CA at a low dosage (150 mg/kg b.w.) modulated the relative abundance of those microbes to be similar to that in the N group. Compared with the N group, MHB and NHB supplementation significantly increased the relative abundance of *Bifidobacterium* and *Akkermansia*.

Furthermore, the 50 most abundant genera presented in the heat map were investigated to identify specific gut microbial taxa that accounted for the most remarkable differences. As shown in Figure 6H, the dominant bacteria in the PB, CA, and PB + CA groups were both relatively similar to and different from those in the M group. The M group exhibited a predominance of *Enterococcus* and *Synechococcus*, while the N group exhibited a predominance of *Desulfovibrio*, *Ruminococcus*, *Mucispirillum*, *Arthrobacter*, *Anaerotruncus*, *Odoribacter*, and *Anaerofustis*. The PB groups exhibited a predominance of *Lactococcus*, *Streptococcus*, *Turicibacter*, and *Clostridum*, and the CA groups exhibited a predominance of *Carnobacterium*, *Olsenella*, and *Paenisporosarcina*. Notably, the MHB group exhibited a predominance of *Akkermansiaceae* and *Bacteroides*, which is considered a next-generation probiotic. The group treated with the combination of PB and CA at a low dosage exhibited a predominance of *Lactobacillus, Lactococcus*, and *Roseburia*. Consequently, these bacteria can serve as biomarkers for the treatment of mice with PB and CA and can act as intestinal indicators for improving hyperlipidemia induced by high-density lipoprotein cholesterol.

### 3.6. Correlation among Gut Microbiota and Hyperlipidemic Parameters in Mice

**Figure 7:**
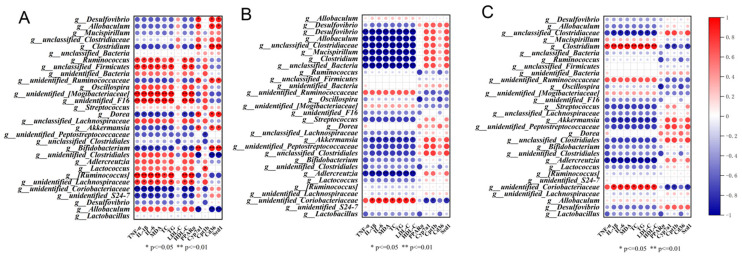
Spearman correlation analysis of top 30 genera with hyperlipidemia-related parameters (**A**) procyanidin B1, (**B**) coumaric acid, and (**C**) combination of procyanidin B1 and coumaric acid (* *p* < 0.05 and ** *p* < 0.01 denote significance).

To determine the potential role of specific intestinal microbial genera whose increased abundance is induced by PB, CA, and PB + CA in mitigating hyperlipidemia, Spearman correlation analysis was adopted to reveal the correlation between the abundance of these dominant genera and some key parameters induced by an HFD (Figure 7). In the phylum Firmicutes, the genera *g_unclassified_Firmicutes*, *g_Ruminococcaceae*, and *g_unidentified_Lachnospiraceae* were positively correlated with TNF-α, IL-1β, IL-6, MDA, TC, TG, and LDL-C and negatively correlated with *Cd36* and *Scd1* gene expression. In the phylum Desulfobacterota, the genus *g_Desulfovibrio*, belonging to the family *Desulfovibrionaceae*, was negatively correlated with TNF-α, IL-1β, IL-6, MDA, TC, TG, PPARα, Cyp7a1, Cpt1b, Cd36, and Scd1. In the phylum Verrucomicrobia, the genus *g_Akkermansia*, belonging to the family *Akkermansiaceae*, was negatively correlated with approximately all HFD-induced parameters; thus, the increased *g_Akkermansia* abundance caused by whole-grain highland barley might contribute to alleviating hyperlipidemia. Similarly, the genera *g_Lactobacillus* and *g_Clostridium* were negatively correlated with the HFD-induced parameters; therefore, the increase in their abundance exhibits a potential benefit for alleviating hyperlipidemia. Thus, supplementation with PB, CA, and PB + CA can reduce the abundance of *g_Ruminococcaceae* and increase that of *g_Desulfovibrio*, *g_Akkermansia*, *g_Lactobacillus*, and *g_Clostridium*, effectively improving the hyperlipidemia phenotype, promoting fat catabolism, and inhibiting fat anabolism.

## 4. Discussion

The liver serves as a major organ for the metabolism of fatty acids and glucose. Previous reports have shown that the liver exhibits the highest expression of PPARα compared with other organs, which is beneficial to the reduction in “atherogenic lipids”, including triglycerides and LDL-C, and the enhancement in plasma HDL-C levels [14,32,33,34]. The results of this work show that supplementation with PB and CA significantly improved the protein and gene expression of PPARα in liver cells. Consistently, the serum contents of TG and LDL-C were decreased, and the serum content of HDL-C was increased. Similarly, Deng et al. reported that the supplementation of a whole-grain highland barley diet significantly diminished the levels of TC and TG [13]. Yao et al. also reported that polyphenol extract from highland barley could decrease the TG and LDL-C contents in HepG2 cells [14]. Therefore, procyanidin B1 and coumaric acid should be assessed as active dietary phenolic compounds from whole-grain highland barley that could alleviate hyperlipidemia in vivo.

Furthermore, one of the most promising candidate genes for determining an individual’s response to dietary or pharmacological cholesterol-lowering interventions is the gene encoding the cytochrome P450 (CYP) enzyme, cholesterol 7α-hydroxylase (*CYP7A1*), which serves as a rate-limiting enzyme in the biosynthesis of bile acids [6,7]. The results of this work show that supplementation with PB and CA significantly up-regulated the protein and gene expression of Cyp7a1. On the other hand, CPT1 facilitates the transport of fatty acids into the mitochondria and participates in the β-oxidation of fatty acids. The supplementation with PB and CA significantly increased the protein and gene expression of Cpt1b in this study. Consistently, previous research also shows that procyanidin B1 significantly activates the gene expression of Cpt1 and thus shows beneficial effects on lipid metabolism [35]. Therefore, these results suggest that PB and CA in whole-grain highland barley may attenuate hyperlipidemia by targeting the expression of Cyp7a1 and Cpt1b, thereby activating the expression of PPARα, thus leading to the regulation of cholesterol homeostasis. Similarly, procyanidin B1 from pine lark extract has been reported to increase the mRNA expression of PPARα and CPT1 in the liver [35]. Moreover, here, the supplementation with PB and CA also showed the significant down-regulation of *Scd1* and *Cd36* gene expression, indicating that those compounds may also alleviate hyperlipidemia by targeting *Scd1* and *Cd36* gene expression, thus reducing hepatic lipogenesis. Notably, the combination of PB and CA significantly up-regulated the gene and protein expression of Cyp7a1, Cpt1b, and PPARα, as well as the gene expression of *Cd36*, thus leading to a decrease in the TG and LDL-C contents and an increase in the HDL-C content. These results indicate that a combination of PB and CA appears to have a synergistic effect on reducing hyperlipidemia by targeting PPARα-mediated hepatic steatosis and lipogenesis, but the related mechanisms need further research. The results may also provide importance for explaining how a whole-grain diet can achieve the amelioration of hyperlipidemia and regulate the hepatic lipid metabolism.

Previous research also shows that PPARα may counter inflammation. Dubois et al. revealed that NF-kB-driven cytokines such as TNF-α, IL-1β, and IL-6 affected the expression of PPARα [36]. In TNFα-treated male Sprague Dawley rats, PPARα mRNA and the corresponding PPARα protein levels were significantly reduced [1]. In this work, supplementation with PB and CA significantly decreased the TNF-α, IL-1β, and IL-6 contents in the serum and increased the protein and gene expression of PPARα, suggesting that PB and CA from whole-grain highland barley may help attenuate hyperlipidemia by reducing inflammation. Similarly, Tie and Su et al. reported that proanthocyanidins such as procyanidin B1, B2, and B4 ameliorated the lipid metabolism by inhibiting inflammation [37,38]. The pathway of PB and CA achieved an anti-inflammatory effect and the relationship with the regulation of hyperlipidemia will be included in our future study.

Extensive evidence indicates that the composition of the gut microbiota can be modulated through interactions among dietary components, thereby positively influencing the prevention and treatment of hyperlipidemia [39,40]. It has been reported that Firmicutes, including genera such as *Ruminococcaceae* and *Lachnospiraceae*, can promote the development of obesity and diabetes in mice [41]. In this work, the relative abundance of *Ruminococcaceae* was significantly decreased in the CA-supplemented mice and was negatively correlated with the HFD-induced serum lipid parameters in the PB, CA, and PB + CA supplementation groups. It has been reported that *Desulfovibrio*, a member of the Desulfobacterota, produces endotoxins and is involved in the pathogenesis of inflammatory bowel diseases [42]. *Lactobacillus* was reported to hold great significance in inhibiting obesity and lipid metabolism and HFD feeding induced a lower abundance of *Lactobacillales* [40,41,43]. In this work, HFD feeding significantly decreased the relative abundance of *Lactobacillus* and *Desulfovibrio*, while the supplementation with PB, CA, and PB + CA significantly increased their abundance. Consistently, the abundance of these bacteria was negatively correlated with the parameters of serum inflammation and lipid disorder, suggesting that PB, CA, and their combination alleviate hyperlipidemia by targeting the bacteria of *Ruminococcaceae*, *Lactobacillus*, and *Desulfovibrio*, thereby modulating gut dysbiosis. Similarly, Cui et al. reported that berberine could increase the abundance of *Desulfovibrio*, thereby exerting hyperlipidemia and anti-inflammatory effects [42]. Duan et al. reported that supplementation with flavonoids from whole-grain oat increased *Desulfovibrio* abundance, which may induce anti-lipogenesis and the inhibition of bile acid reabsorption [2]. It is noteworthy that within the phylum *Verrucomicrobia*, *Akkermansia* has been demonstrated to be inversely associated with obesity, diabetes, and several other intestinal disorders [19,44,45]. In this work, the relative abundance of *Akkermansia* in the WHB and NHB groups was significantly higher than in other groups. Furthermore, the heatmap analysis showed that the relative abundance of *Akkermansia* showed negative correlations with the serum TC, TG, and LDL-C contents and positive correlations with PPARα, Cyp7a1, and Cpt1b expression in the PB + CA supplementation group, indicating that whole-grain highland barley may modulate hyperlipidemia by regulating *Akkermansia* and that the combination of PB and CA may target *Akkermansia* and exhibit similar effects on alleviating hyperlipidemia to whole-grain highland barley. Consistently, Li et al. reported that whole-grain highland barley significantly increased *Akkermansia* levels [20]. Hou et al. also reported that whole mung bean supplementation significantly increased the abundance of *Akkermansia* [29]. However, the difference between PB, CA and PB + CA supplementation on modulating intestinal microbiota dysbiosis needs further exploration.

## 5. Conclusions

In this study, PB and CA from whole-grain highland barley were found to be the active phenolic compounds that alleviate hyperlipidemia through the activation of PPARα expression, resulting in an increase in the serum HDL-C levels and reduction in the TC, TG, and LDL-C levels. Consistent with the increased PPARα expression, PB and CA displayed anti-inflammatory effects via decreasing the serum TNF-α, IL-1β, and IL-6 contents. Moreover, PB and CA supplementation modulated the relative abundance of the gut microbiota, such as *Ruminococcaceae*, *Lactobacillus*, *Desulfovibrio*, and *Akkermansia*, and showed a significant correlation with the inflammation and lipid metabolism parameters, indicating that an improvement in gut microbiota dysbiosis may also help in alleviating hyperlipidemia. Interestingly, the combination of PB and CA showed a synergistic effect on targeting PPARα, thereby alleviating hyperlipidemia, and the relative abundance of the microbiota of *Akkermansia* was significantly increased, which was consistent with the supplementation of whole-grain highland barley. These results help explain the relative mechanism of dietary polyphenols from whole-grain highland barley in hyperlipidemia, where the use of these compounds could be considered an effective strategy to combat this condition.

## Data Availability

The original contributions presented in the study are included in the article and further inquiries can be directed to the corresponding author.
